# Dendrimer-Functionalized Laponite Nanodisks as a Platform for Anticancer Drug Delivery

**DOI:** 10.3390/nano5041716

**Published:** 2015-10-20

**Authors:** Rania Mustafa, Yu Luo, Yilun Wu, Rui Guo, Xiangyang Shi

**Affiliations:** 1 College of Chemistry, Chemical Engineering and Biotechnology, Donghua University, Shanghai 201620, China; E-Mails: raniamustafaa@gmail.com (R.M.); luoyuboy@126.com (Y.L.); zodiac.bio@gmail.com (Y.W.); 2 CQM-Centro de Química da Madeira, Universidade da Madeira, Campus da Penteada, 9000-390 Funchal, Portugal

**Keywords:** drug delivery system, laponite, PAMAM dendrimer, anticancer efficacy

## Abstract

In this study, we synthesized dendrimer-functionalized laponite (LAP) nanodisks for loading and delivery of anticancer drug doxorubicin (DOX). Firstly, LAP was modified with silane coupling agents and succinic anhydride to render abundant carboxyl groups on the surface of LAP. Then, poly(amidoamine) (PAMAM) dendrimer of generation 2 (G2) were conjugated to form LM-G2 nanodisks. Anticancer drug DOX was then loaded on the LM-G2 with an impressively high drug loading efficiency of 98.4% and could be released in a pH-sensitive and sustained manner. Moreover, cell viability assay results indicate that LM-G2/DOX complexes could more effectively inhibit the proliferation of KB cells (a human epithelial carcinoma cell line) than free DOX at the same drug concentration. Flow cytometry analysis and confocal laser scanning microscope demonstrated that LM-G2/DOX could be uptaken by KB cells more effectively than free DOX. Considering the exceptional high drug loading efficiency and the abundant dendrimer amine groups on the surface that can be further modified, the developed LM-G2 nanodisks may hold a great promise to be used as a novel platform for anticancer drug delivery.

## 1. Introduction

Cancer is one of the most significant threats to human health and will cause about 15 million deaths in 2020 [1]. As a conventional treatment, chemotherapeutic drugs have some inevitable disadvantages, such as poor water solubility and stability, short circulation time, and severe side-effects to normal tissues [2,3]. With the development of nanotechnology, different kinds of nanocarriers, such as dendrimers [4], carbon nanotubes [5], and micelles [6], have been used to load anticancer drugs and display an enhanced accumulation in tumor tissues rather than normal cells *via* enhanced permeability and retention (EPR) effects [6,7,8,9]. Thus, developing a novel and functional drug delivery systems has been regarded as an effective way to enhance the therapeutic efficiency of anticancer drugs.

Laponite (LAP) is a kind of disc-shaped synthetic clay with a diameter of 25 nm and thickness of 1 nm, and shares a similar composition and structure to the natural clay mineral hectorite. Recently, LAP has attracted more and more attention in drug delivery [10,11,12] due to its physiological stability and impressively high specific surface area (370 m^2^·g^−1^) [13]. For example, LAP can load doxorubicin (DOX) with a payload as high as 98.3% and display a sustained release of DOX in a pH-dependent manner, and the formed LAP/DOX systems display a better therapeutic efficiency than free DOX [14]. After modified with folic acid or lactobionic acid as targeting agents *via* the linkage of silane coupling agents, LAP-based drug delivery systems could specifically deliver anticancer drugs to tumor cells and enhance the inhibition effect of cancer cells [15,16]. However, this kind of surface modification may cause a decrease in drug loading efficiency. Therefore, to improve the performance of a LAP-based drug delivery system, it is a great challenge to endow LAP with abundant functional groups on the surface for further targeting modification without sacrificing its inherent loading capacity.

Poly(amidoamine) (PAMAM) dendrimers are a class of synthetic macromolecules with a low polydispersity index and branched interiors [17,18]. The internal hydrophobic cavities of dendrimers and abundant functional groups on the surface make them an ideal carrier system for anticancer drug loading and targeted delivery [4,19,20,21]. Many anticancer drugs, such as methotrexate (MTX) [22], 5-fluorouracil (5-FU) [23], and DOX [4,24], have been effectively loaded by PAMAM dendrimers *via* different mechanisms including electrostatic interaction, physical encapsulation, and covalent conjugation. Dendrimer-based drug delivery systems have been proven to increase the water solubility of anticancer drugs, prolong their circulation time, and passively target tumor tissues through EPR effect. Moreover, PAMAM dendrimers have abundant amine groups on the surface for further modification with targeting agents for specific delivery of anticancer drugs. In our previous work, PAMAM dendrimer was used to target human liver cancer cells by covalent linking with lactobionic acid (LA) as a liver cancer cell targeting agent onto partially-acetylated generation 5 PAMAM dendrimers [25]. In another work, folic acid-modified dendrimer-MTX conjugate was found to be much more effective than free MTX as well as dendrimer-MTX conjugate due to the specific delivery of anticancer drug to tumors [22]. Biocompatible poly(ethylene glycol) (PEG) can also be modified on dendrimers to decrease the cytotoxicity of dendrimers and improve their biodistribution behavior [26,27]. Since the combination of organic and inorganic nanohybrids has been demonstrated to have a synergistic performance [28,29,30], it is plausible to hypothesize that the modification of PAMAM dendrimers on the surface of LAP nanodisks would improve the performance of a LAP-based drug delivery system through providing additional drug loading capacity and endowing LAP nanodisks with active functional groups for targeting modification.

In this study, LAP nanodisks were silanized to have amine groups and then modified with succinic anhydride (SAH) to render them with abundant carboxyl groups on the surface, and finally conjugated with amine-terminated PAMAM dendrimers of generation 2 (G2). The formed dendrimer-modified LAP (LM-G2) nanodisks were characterized by thermogravimetric analysis (TGA), Fourier transform infrared (FTIR) spectroscopy, zeta potential, and dynamic light scattering (DLS). Then, the anticancer drug DOX was encapsulated into the LM-G2 and the release profile of DOX from the LM-G2/DOX complexes was investigated under different pH conditions. Finally, *in vitro* anticancer efficacy of the LM-G2/DOX complexes was explored *via* 3-(4,5-dimethylthiazol-2-yl)-2,5-diphenyltetrazolium bromide (MTT) assay, and the cellular uptake of the LM-G2/DOX complexes was investigated by confocal laser scanning microscopy (CLSM) observation and flow cytometric (FCM) analysis. To the best of our knowledge, this is the first report related to the modification of dendrimers onto LAP nanodisks for the efficient delivery of anticancer drugs.

## 2. Results and Discussion

### 2.1. Synthesis and Characterization of LM-G2

**Figure 1:**
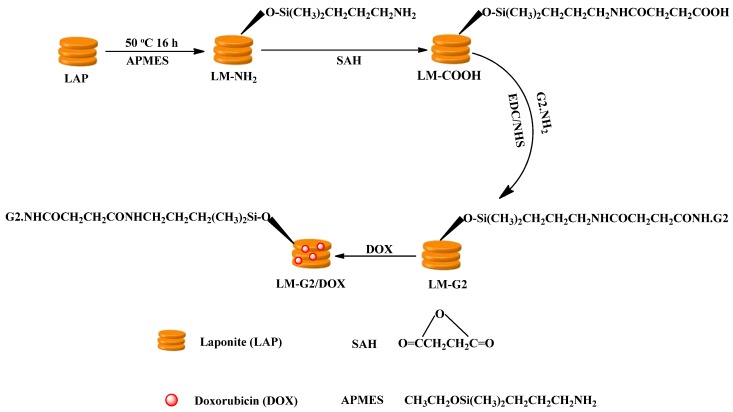
Schematic representation of the preparation of the formed dendrimer-modified LAP (LM-G2)/doxorubicin (DOX) complexes.

In this study, G2 PAMAM dendrimer was designed to chemically conjugate with LAP in order to introduce active functional groups on the surface of LAP and improve the drug loading ability as well (Figure 1). Firstly, amino-propyldimethylethoxysilane (APMES) was modified on the surface of LAP nanodisks to render them with amine surface groups, and SAH was reacted with the LAP surface amines to render the nanodisks with abundant carboxyl groups on the surface. Then G2 PAMAM dendrimers were chemically linked onto the LAP through the formation of amide groups between carboxyl groups of LAP and the amine groups of G2 with the aid of 1-(3-dimethylaminopropyl)-3-ethylcarbodiimide (EDC)/N-Hydroxysuccinimide (NHS). Finally, the anticancer drug DOX was encapsulated to form the LM-G2/DOX complexes.

**Figure 2:**
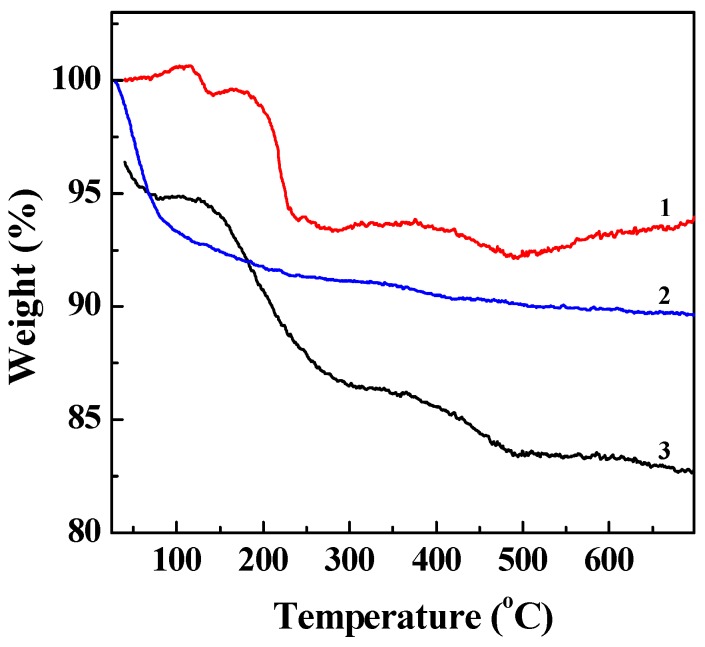
Thermogravimetric analysis (TGA) curves of laponite (LAP) (1), LM-NH_2_ (2), and LM-G2 (3).

In order to demonstrate the modification of APMES and G2 on the LAP nanodisks, LM-G2 nanodisks were characterized by TGA (Figure 2). Compared with pristine LAP, the weight loss of LM-NH_2_ in the temperature range from 200 to 600 °C was 1.67%, indicating that 1.67% APMES has been modified on the surface of LAP. Furthermore, by subtracting the residue weight of LM-NH_2_ at 600 °C, LM-G2 displayed about 5.13% weight loss, which may be ascribed to the amount of G2 dendrimer modified onto LAP nanodisks (This means that 51.3 μg of G2 dendrimers were conjugated onto each milligram of LAP). Therefore, the TGA results suggest that LM-NH_2_ and LM-G2 have been successfully synthesized. It should be noted that the weight loss of LM-NH_2_ and LM-G2 at the temperature from ~25 to 60 °C is due to the absorbed moisture. In our calculation, we have excluded the contribution of the moisture.

The changes of surface potential and hydrodynamic size during LAP modification were monitored by DLS (Table 1). Pristine LAP has a negative surface potential of −32.5 ± 0.5 mV, and after silanization, zeta potential of LM-NH_2_ increased to −18.6 ± 1.8 mV. This may be due to the fact that amine groups modified by silanization could partly shield the negative charges on the surface of LAP. After the subsequent reaction with SAH, the zeta potential decreased to −19.6 ± 2.5 mV. When the G2 dendrimer was finally conjugated, LM-G2 displays a positive potential of 5.8 ± 0.5 mV, indicating the successful modification of amine-terminated G2 dendrimer on the LAP surface. Meanwhile, the hydrodynamic diameter of LAP nanodisks increased during modification. The silanization step enabled a size increase from 74 ± 8.9 nm (LAP) to 225 ± 12.7 nm (LM-NH_2_), suggesting a certain degree of aggregation of the nanodisks. And after conjugation with G2 dendrimers, the obtained LM-G2 shows a size of 869 ± 18.2 nm. This suggests that G2 may not only modify the surface of a single nanodisk, but also act as bridge among several LAPs, resulting in a cluster structure in solution. It seems that the clustered nanodisks do not compromise the narrow size distribution of the particles, presumably due to the fact that the formed clusters are rather uniform in size. The changes of surface potential demonstrated the step-by-step modification as designed and the successful formation of the LM-G2.

**Table 1:** Zeta-potential and hydrodynamic size of the LAP, LM-NH_2_, LM-COOH, and LM-G2.

Materials	Zeta Potential (mV)	Hydrodynamic Size (nm)
LAP	−32.5 ± 0.5	74 ± 8.9
LM-NH_2_	−18.6 ± 1.8	225 ± 12.7
LM-COOH	−19.6 ± 2.5	566 ± 5.3
LM-G2	+5.8 ± 0.5	869 ±18.2

**Figure 3:**
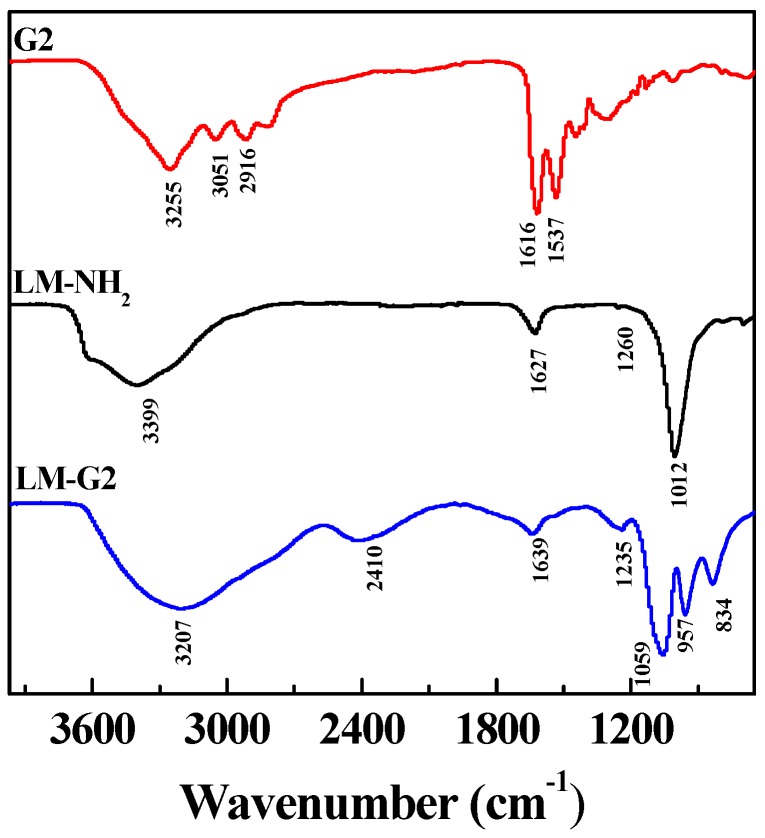
Fourier transform infrared (FTIR) spectra of generation 2 (G2), LM-NH_2_, and LM-G2.

FTIR was also used to characterize the LM-NH_2_, G2 dendrimer, and LM-G2 (Figure 3). In the spectrum of LM-NH_2_, the band at 1012 cm^−1^ can be assigned to the irregular stretching vibration of the Si-O bond, suggesting the successful silanization reaction [15,31]. After the conjugation of G2, the broad peak at 3207 cm^−1^ and a shoulder peak at 2916 cm^−1^ in the spectrum of LM-G2 can be assigned to the N–H stretching vibration of amide groups and –CH_2_– stretching vibration of methylene groups in the dendrimer structure, and the peak at 957 cm^−1^ represents the N–H and C–H wagging vibrations [32,33]. The peak at 1639 cm^−1^ could be assigned to the amide bond formed between the LAP carboxyl and the dendrimer terminal amines. Therefore, FTIR results were in good agreement with DLS results, confirming the successful formation of the LM-G2.

### 2.2. Loading Efficiency of DOX into LM-G2 Nanodisks

**Figure 4:**
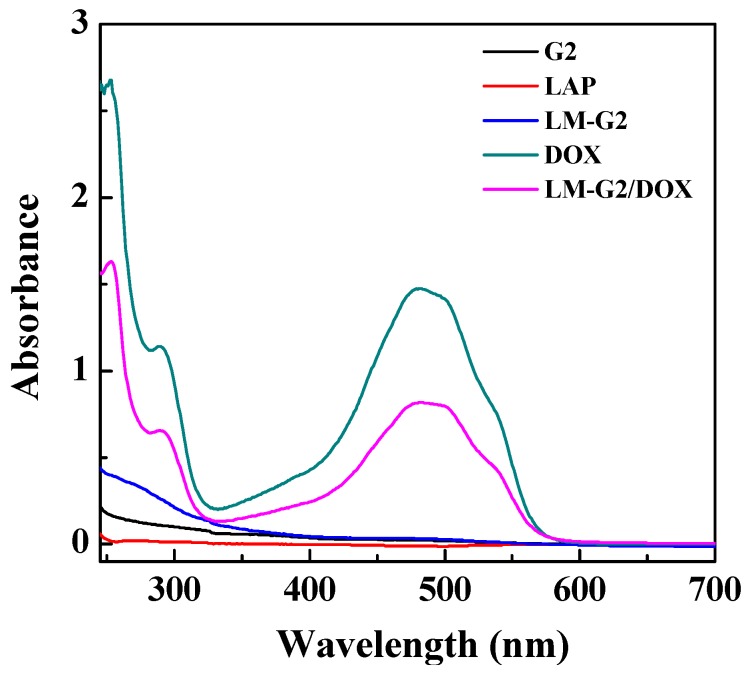
UV-Vis spectra of G2, laponite (LAP), LM-G2, free DOX, and LM-G2/DOX.

The encapsulation of DOX within the LM-G2 was confirmed by UV-Vis spectrometry, and the result is shown in Figure 4. It can be clearly seen that there is no significant absorption peak in the spectra of LAP, G2, and LM-G2. In contrast, after the encapsulation of DOX, LM-G2/DOX complexes display an obvious absorption peak at 480 nm, indicating the successful encapsulation of DOX [14,15]. The drug loading efficiency of LM-G2 was calculated to be 98.4%, which is much higher than that of LM-NH_2_/DOX complexes (87.6%) in our previous work [15]. This result demonstrated that G2 dendrimer modified on the surface of LAP may increase the drug loading capacity of LM-G2 due to the additional loading capacity provided by the attached dendrimers.

### 2.3. Release Profile of DOX from LM-G2/DOX Complexes

**Figure 5:**
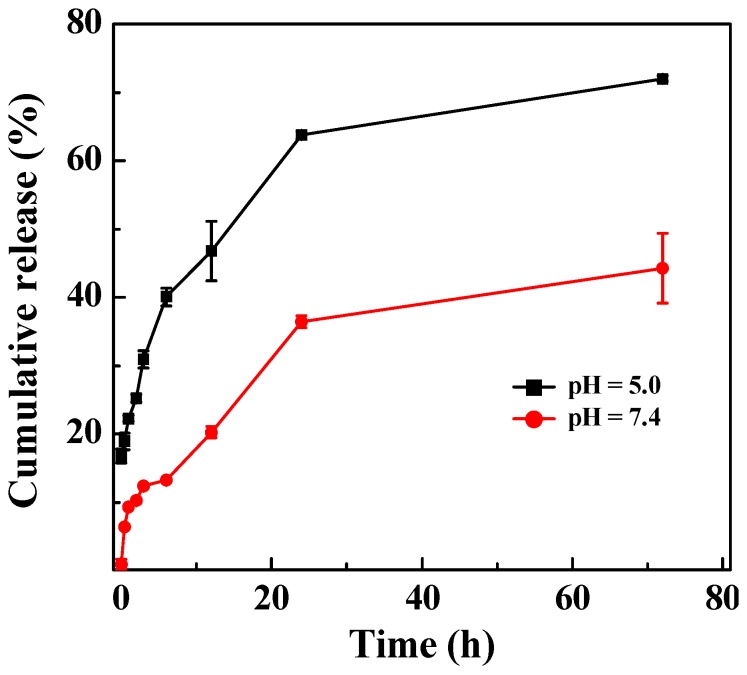
*In vitro* release of DOX from the LM-G2/DOX nanodisks at 37 °C under different pH conditions (pH = 5.0 and 7.4).

The cumulative release of DOX from the LM-G2/DOX complexes was investigated under both pH 5.0 and 7.4 conditions (Figure 5). Clearly, DOX can be released from the LM-G2/DOX complexes gradually under both pH circumstances with different release rates. Within 72 h, about 72% of DOX were released from LM-G2/DOX complexes under pH 5.0, while only 44% of DOX were released under pH 7.4. This result indicated that the release of DOX from the LM-G2/DOX complexes was in a pH-dependent sustained manner, with a higher release rate at an acidic pH condition. The pH-dependent release of DOX from LM-G2/DOX nanodisks is likely attributed to the different hydrophilicity of the DOX drug under different pH conditions. Under an acidic pH (pH = 5.4) condition, the DOX·HCl maintains a salt form and is quite soluble in water, which affords the fast release from the LAP interlayer space. In contrast, under a physiological pH condition (pH = 7.4), the DOX·HCl is able to be deprotonated to form a hydrophobic neutral molecule. Therefore, the DOX release rate from LM-G2/DOX is much higher under an acidic pH condition than under a physiological pH condition, in agreement with previous reports [14,34,35]. Given that the microenvironment of tumors is acidic, the pH-dependent release profile of the LM-G2/DOX complexes is beneficial for tumor therapy and may lower the side effect to normal cells in physiological environment. More interestingly, when compared with LAP/DOX in our previous work, which can only release about 29.5% of the loaded DOX at an acidic condition after nine days [14], LM-G2/DOX complexes showed a much higher release rate and achieved a higher accumulative release percentage. This fast and more complete release of DOX in an acidic environment might be due to the electrostatic repulsion between the protonated DOX and the protonated G2 dendrimers modified on the LAP nanodisks.

### 2.4. Antitumor Efficacy of the LM-G2/DOX Complexes

**Figure 6:**
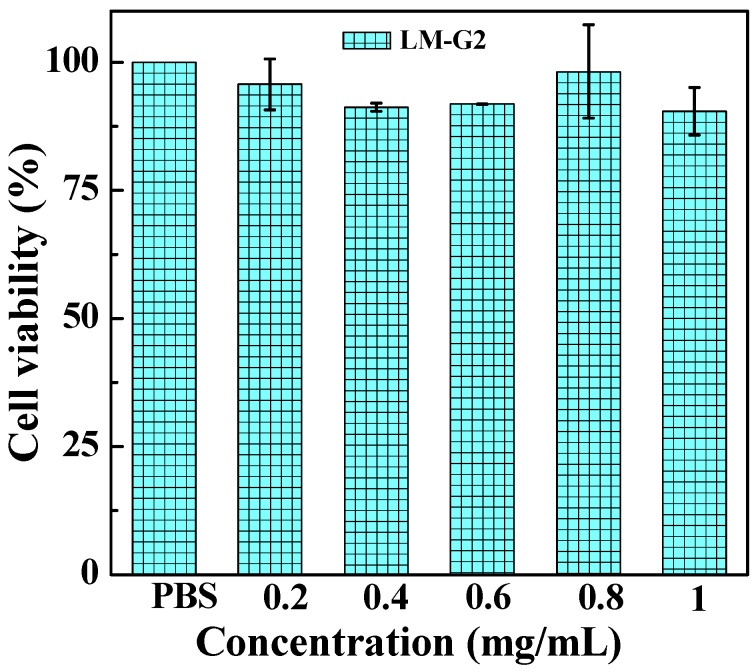
Viability of KB cells treated with LM-G2 nanodisks at a concentration of 0.2, 0.4, 0.6, 0.8, and 1.0 mg/mL for 24 h at 37 °C.

**Figure 7:**
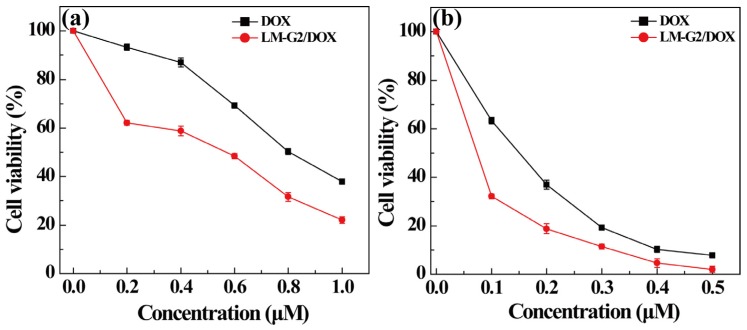
*In vitro* MTT cell viability assay of KB cells treated with LM-G2/DOX and free DOX at different DOX concentrations for (**a**) 24 h and (**b**) 48 h.

The cytotoxicity of the LM-G2 nanodisks and the antitumor activity of the LM-G2/DOX complexes were evaluated *via* MTT colorimetric assay. Figure 6 shows that after incubation with LM-G2 for 24 h, the viability of KB cells kept over 80% within the given concentration range, indicating the good cytocompatibility of LM-G2 nanodisks. In Figure 7, LM-G2/DOX could significantly inhibit the proliferation of KB cells in a dose-dependent manner, which is similar to free DOX. The half maximal inhibitory concentration (IC_50_) values of LM-G2/DOX complexes were 569.75 nM for 24 h treatment and 73.69 nM for 48 h treatment, which were 1.41 times and 2.04 times lower than that of free DOX (803.27 nM for 24 h, 150.57 nM for 48 h). Therefore, LM-G2/DOX complexes exhibited much higher antitumor activity than free DOX at the same drug concentration after 24 h or 48 h of incubation.

**Figure 8:**
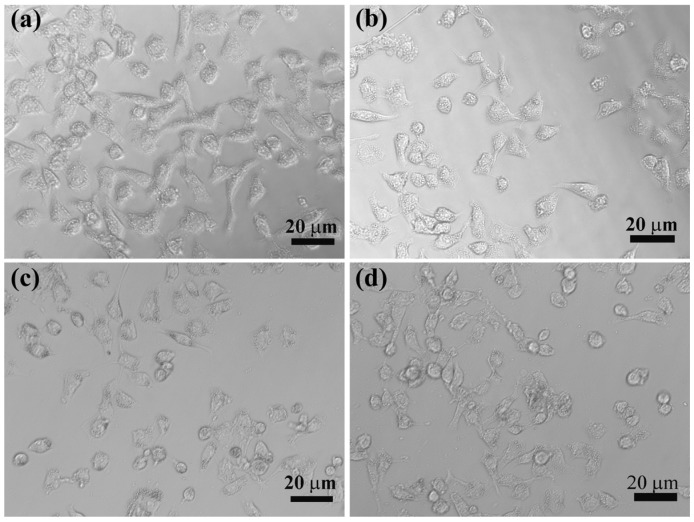
Phase contrast photomicrographs of KB cells treated with (**a**) PBS, (**b**) LM-G2, (**c**) free DOX, and (**d**) LM-G2/DOX at a DOX concentration of 0.2 μM for 24 h.

To observe the cell morphology, KB cells treated with LM-G2, free DOX, and LM-G2/DOX complexes at the same DOX concentration were observed by phase contrast microscopy (Figure 8). KB cells treated with LM-G2 show similar morphology to those treated with PBS, indicating the low cytotoxicity of LM-G2 as nanocarriers. In contrast, the morphology of KB cells treated with LM-G2/DOX complexes were rounded in shape and detached from the bottom of the plate, indicative of the cells that have undergone apoptosis. Therefore, the observation of cell morphology demonstrated that LM-G2/DOX complexes could inhibit the proliferation of KB cells effectively, consistent with the MTT assay results.

### 2.5. Intracellular Uptake of LM-G2/DOX Complexes

**Figure 9:**
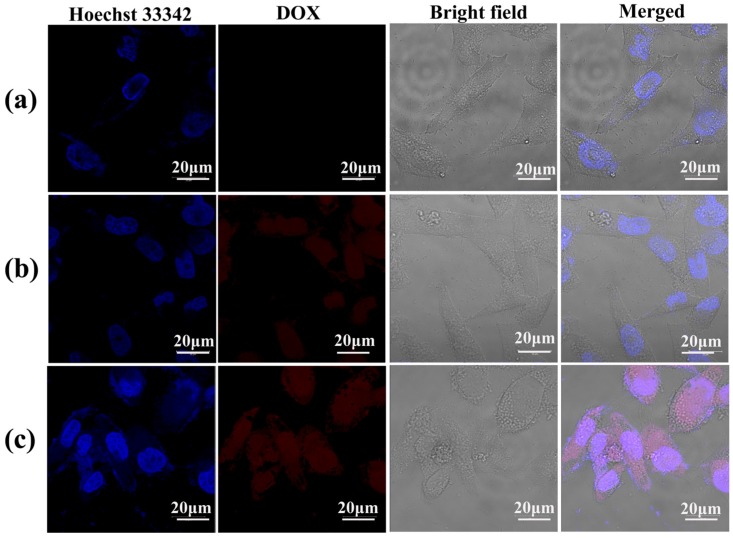
Confocal laser scanning microscopy (CLSM) images of KB cells treated with (**a**) PBS (control), (**b**) free DOX and (**c**) LM-G2/DOX nanodisks with a DOX concentration of 0.6 μM for 4 h at 37 °C. KB cells treated with PBS were used as control.

To confirm the intracellular uptake of the LM-G2/DOX complexes, CLSM was used to observe KB cells treated with free DOX and LM-G2/DOX complexes (Figure 9). The cell nuclei were counterstained with Hoechst 33342 in blue, and KB cells treated with PBS were set as control. After 4 h of incubation with free DOX and LM-G2/DOX complexes, both KB cells exhibited red fluorescence signals associated with DOX molecules in cell nuclei and cytoplasm, while control cells treated with PBS did not display red fluorescence signals. More importantly, KB cells incubated with LM-G2/DOX complexes displayed more red dots than those treated with free DOX in the cell nuclei, suggesting the internalization of LM-G2/DOX complexes, instead of the released free DOX. The dendrimers modified on the LAP surface may facilitate the uptake of LM-G2/DOX complexes by KB cells likely *via* phagocytosis and diffusion *via* cell walls [36,37].

**Figure 10:**
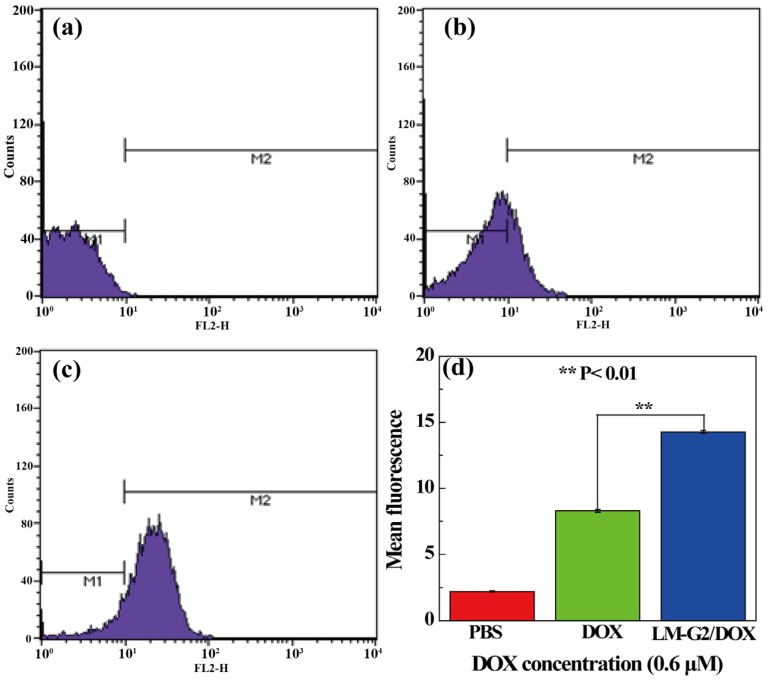
Flow cytometric (FCM) analysis of the *in vitro* cellular uptake of KB cells treated with (**a**) PBS (control), (**b**) free DOX and (**c**) LM-G2/DOX nanodisks for 2 h; (**d**) shows the comparison of the mean fluorescence of cells treated by different materials (**, *p* < 0.01).

To further quantitatively verify the intracellular uptake of the LM-G2/DOX complexes, FCM was performed to analyze the mean fluorescence of KB cells after treated with free DOX and LM-G2/DOX complexes at a DOX concentration of 0.6 μM for 2 h (Figure 10). Compared with KB cells treated with PBS as control, an apparent enhancement of mean fluorescence can be observed in free DOX and LM-G2/DOX groups, indicating the cellular uptake of free DOX and LM-G2/DOX by KB cells. Furthermore, the mean fluorescence of KB cells treated with LM-G2/DOX was about 1.75 times higher than that of cells treated with free DOX (*p* < 0.01). This result demonstrated that, in contrast to free DOX, LM-G2/DOX complexes could be more efficiently uptaken by KB cells and deliver DOX to cancer cells, which is consistent with CLSM imaging data. Therefore, the LM-G2 nanodisks could be a promising and effective carrier in anticancer drug delivery.

## 3. Experimental Section

### 3.1. Materials

LAP and DOX·HCl were purchased from Zhejiang Institute of Geology and Mineral Resources (Hangzhou, China) and Beijing Huafeng Pharmaceutical Co. Ltd. (Beijing, China), respectively. Ethylenediamine core G2 PAMAM dendrimers (*M*w = 3256) were supplied from Dendritech (Midland, MI, USA). 1-Ethyl-3-(3-dimethylaminopropyl) carbodiimide hydrochloride acid (EDC) was purchased from J&K Chemical Ltd (Shanghai, China). Triethylamine, NHS, DMSO, APMES, MTT and Hoechst 33342 and all the other chemicals and solvents were purchased from Aldrich (St. Louis, MO, USA). KB cells were obtained from the Institute of Biochemistry and Cell Biology (Chinese Academy of Sciences, Shanghai, China). Roswell Park Memorial Institute-1640 (RPMI-1640) medium, fetal bovine serum (FBS), penicillin, and streptomycin were from Hangzhou Jinuo Biomedical Technology (Hangzhou, China). All of the cell culture flasks and plates were from NEST Biotechnology (Shanghai, China). Water used in all experiments was purified using a Milli-Q Plus 185 water purification system (Millipore, Bedford, MA, USA) with a resistivity higher than 18 MΩ·cm.

### 3.2. Synthesis of LM-G2 Nanodisks

LAP powder (75 mg) was dispersed in 60 mL of water at 50 °C overnight under magnetic stirring to form an LAP aqueous solution (1.25 mg/mL). An APMES (2%, 2 mL) aqueous solution was added dropwise to 5 mL of LAP solution under vigorous stirring at 50 °C water bath for 16 h. Then the reaction mixture was dialyzed against water (12 times, 2 L for each time) for three days using a dialysis membrane with a molecular weight cut-off (MWCO) of 14,000. The obtained LM-NH_2_ aqueous solution was stored at 4 °C before use. The concentration of LM-NH_2_ was verified by lyophilizing a portion of LM-NH_2_ with a given volume.

To synthesize the carboxyl-functionalized LAP nanodisks, the above LM-NH_2_ was added with 5 mL DMSO solution containing 20.0 mg of SAH with a molar ratio of SAH/LM-NH_2_ = 2.1:1 under vigorous stirring and reacted for 24 h. After that, the resultant solution was purified by dialysis against water (12 times, 2 L for each time) for three days using a dialysis membrane with an MWCO of 14,000. Then LM-COOH (6.0 mg, 5 mL) solution was mixed with EDC (7.0 mg) and NHS (4.0 mg) in 5 mL PBS for 3 h to active the carboxyl groups. Finally, the activated LM-COOH solution was dropwise added into G2 solution (2 mg/mL, 5 mL) and stirred for 3 days at room temperature. The reaction mixture was dialyzed against PBS (three times, 2 L) and water (three times, 2 L) using a dialysis bag with an MWCO of 14,000 for three days.

### 3.3. Characterization

TGA was carried out using a TG209F1 (NETZSCH Instruments Co., Ltd, Bavaria, Germany) thermo-gravimetric analyzer. The samples were heated from room temperature to 700 °C at a rate of 10 °C/min under a nitrogen atmosphere. Zeta potential and DLS measurements were carried out using a Malvern Zetasizer Nano ZS model ZEN3600 (Worcestershire, UK) equipped with a standard 633 nm laser. FTIR spectrometry was performed using a Nicolet Nexus 670 FTIR spectrometer (Nicolet-Thermo, Waltham, MA, USA). All spectra were recorded using a transmission mode with a wavenumber range of 650–4000 cm^−1^.

### 3.4. Loading of DOX within the LM-G2 Nanodisks

An aqueous solution of DOX solution (1.25 mg/mL) was prepared, and the LM-G2 concentration was set as 2.70 mg/mL. Equal volume of LM-G2 and DOX solutions were mixed together and magnetically stirred for 24 h. Then the solution was centrifugated (8000 rpm, 5 min) and resuspended in water for 5 times to remove free DOX. Finally, LM-G2/DOX nanodisks were obtained and stored in dark at room temperature. To determine the drug loading efficiency, the supernatants were collected after five times centrifugation, and the free DOX concentration of the supernatants was quantified using a Lambda 25 UV-Vis spectrophotometer (Perkin Elmer, Waltham, MA, USA) at 480 nm with the standard absorbance-concentration calibration curve at the same wavelength. The drug encapsulation efficiency was defined as the ratio of the mass of encapsulated drug to the mass of drug used for encapsulation through the following Equation:

(1) $$Drug\;efficiency\;=\;\frac{Mass\;of\;encapsulated\;DOX}{Total\;mass\;of\;DOX\;used\;in\;encapsulation}\times 100\%$$

### 3.5. In Vitro Drug Release Kinetics

The *in vitro* drug release kinetics of the LM-G2/DOX complexes was investigated under different pH conditions. Briefly, LM-G2/DOX (4.92 mg) dispersed into 1 mL of PBS phosphate buffer saline (pH = 7.4) or 1 mL acetic acid-sodium acetate buffer solution (pH = 5.0) were placed into dialysis bags with an MWCO of 14,000. Then, the dialysis bags were placed into 9 mL of corresponding buffer solutions in sample vials. All the samples were incubated in vapor-bathing constant temperature vibrator at 37 °C. At each predetermined time point, 1 mL of solution was taken out from the outer phase and an equal volume of corresponding buffer was replenished into each sample vial. The DOX concentration of the samples was quantified by UV-Vis spectrometry at 480 nm.

### 3.6. Cell Culture and Cytotoxicity Assay

KB cells were regularly cultured in a 25-mL culture flask with 5 mL of RPMI-1640 medium containing 10% FBS, 100 U/mL penicillin, and 100 U/mL streptomycin in a humidified incubator with 5% CO_2_ at 37 °C. KB cells with a density of 1 × 10^4^ cells per well were seeded into a 96-well plate and cultured overnight to bring the cells to confluence. Then the medium was replaced with fresh medium containing PBS, LM-G2, free DOX, and LM-G2/DOX nanodisks with a final DOX concentration ranging from 0.1 to 1.0 μM. After 24 or 48 h incubation, cells were rinsed with PBS, and then 90 μL fresh medium containing 10 μL MTT was added to each well. After incubation at 37 °C for 4 h, 100 μL of DMSO was added to dissolve the purple MTT formazan crystals. The plates were read at 570 nm using an MK3 Microplate Reader (Thermo, Waltham, MA, USA). Mean and standard deviation for the triplicate wells for each sample were reported. In parallel, before MTT assay, the morphology of cells after treatment for 24 h was observed using a Leica DM IL LED inverted phase contrast microscope with a magnification of 200× for each sample.

### 3.7. FCM Analysis

KB cells with a density of 1 × 10^5^ cells/well were seeded in a 6-well tissue culture plate one day before to bring the cells to confluence. Then the culture medium was discarded and cells were incubated with fresh RPMI-1640 medium containing free DOX or LM-G2/DOX complexes with a final DOX concentration of 0.6 μM for an additional 2 h. After that, the medium were discarded and cells were rinsed with PBS three times. Cells were harvested and suspended in 1 mL of PBS under 4 °C. Cells treated with PBS were used as control. Then, FACS Calibur flow cytometer (Becton Dickinson, Mountain View, CA, USA) equipped with a 15 mW, 488 nm, and air-cooled argon ion laser was used to measure the fluorescence intensity of DOX using FL2 channel. For all samples, 1 × 10^4^ cells were counted, and all the measurements were in triplicate.

### 3.8. CLSM Imaging

The intracellular uptake of DOX was also qualitatively tested by CLSM (Carl Zeiss LSM 700, Jena, Germany). Cover slips with a diameter of 14 mm were pretreated with 5% HCl, 30% HNO_3_, and 75% alcohol before use, and then soaked in the 1640 medium in a 12-well tissue culture plate overnight. 5 × 10^4^ KB cells were seeded into each well and cultured for about 48 h to bring the KB cells to confluence. Before CLSM imaging, PBS, LM-G2/DOX nanodisks and free DOX with a final DOX concentration of 0.6 μM were used to treat KB cells adhered onto the cover slips for 4 h. Then the KB cells were fixed by glutaraldehyde (2.5%) for 15 min at 4 °C and counterstained with Hoechst 33342 (1 μg/mL) for 15 min at 37 °C using a standard procedure. Finally, the samples were imaged using a 63× oil-immersion objective lens.

## 4. Conclusions

In summary, G2 dendrimer-modified LAP nanodisks (LM-G2) were successfully synthesized and used to encapsulate anticancer drug DOX with an exceptional high loading efficiency (98.4%). The formed LM-G2/DOX complexes not only display a pH-dependent sustained release profile, but also release DOX more thoroughly at an acidic pH condition at the same period of time in comparison to LAP/DOX or LM-FA/DOX reported in our previous work. More importantly, LM-G2/DOX could be effectively uptaken by cancer cells and displayed stronger inhibitory effect than free DOX. Therefore, considering the high drug loading efficiency and abundant functional groups on the surface, LM-G2 nanodisks can be used as a versatile delivery platform for various anticancer drugs in cancer therapy.
