# Erratum: Mustafa, R.; Luo, Y.; Wu, Y.; Guo, R.; Shi, X. Dendrimer-Functionalized Laponite^®^ Nanodisks as a Platform for Anticancer Drug Delivery. *Nanomaterials* 2015, *5*, 1716–1731

**DOI:** 10.3390/nano6060114

**Published:** 2016-06-14

**Authors:** Nanomaterials Editorial Office

**Affiliations:** Klybeckstrasse 64, CH-4057 Basel, Switzerland

It has been brought to our attention that Laponite^®^ is a trademark of BYK Additives, however the trademark symbol is missing in [1]. We apologize for an inconvenience caused by this omission. The symbol will be added to the online version of the article.
